# KNN-MDR: a learning approach for improving interactions mapping performances in genome wide association studies

**DOI:** 10.1186/s12859-017-1599-7

**Published:** 2017-03-21

**Authors:** Sinan Abo Alchamlat, Frédéric Farnir

**Affiliations:** Department of Biostatistics, Faculty of Veterinary Medicine, FARAH, University of Liège, Sart Tilman B43, 4000 Liege, Belgium

**Keywords:** Gene-gene interaction, Epistasis, Single nucleotide polymorphism, Genome-wide association study, Multi dimensional reduction, K-nearest neighbors

## Abstract

**Background:**

Finding epistatic interactions in large association studies like genome-wide association studies (GWAS) with the nowadays-available large volume of genomic data is a challenging and largely unsolved issue. Few previous studies could handle genome-wide data due to the intractable difficulties met in searching a combinatorial explosive search space and statistically evaluating epistatic interactions given a limited number of samples. Our work is a contribution to this field. We propose a novel approach combining K-Nearest Neighbors (KNN) and Multi Dimensional Reduction (MDR) methods for detecting gene-gene interactions as a possible alternative to existing algorithms, e especially in situations where the number of involved determinants is high. After describing the approach, a comparison of our method (KNN-MDR) to a set of the other most performing methods (i.e., MDR, BOOST, BHIT, MegaSNPHunter and AntEpiSeeker) is carried on to detect interactions using simulated data as well as real genome-wide data.

**Results:**

Experimental results on both simulated data and real genome-wide data show that KNN-MDR has interesting properties in terms of accuracy and power, and that, in many cases, it significantly outperforms its recent competitors.

**Conclusions:**

The presented methodology (KNN-MDR) is valuable in the context of loci and interactions mapping and can be seen as an interesting addition to the arsenal used in complex traits analyses.

**Electronic supplementary material:**

The online version of this article (doi:10.1186/s12859-017-1599-7) contains supplementary material, which is available to authorized users.

## Background

These last years have seen the emergence of a wealth of biological information. Technical improvements in genotyping and sequencing technologies have facilitated the access to the genome sequence and to massive data on genes expression and on proteins. This large availability of molecular information has revolutionized the research in many fields of biology. In parallel to these technical developments, methodological advances are needed to address the various questions of scientific interest that have been targeted when developing these new molecular tools. For example, the identification of up to several millions genomic variations in many species and the development of chips allowing for an effective genotyping of SNPs panels in large cohorts have triggered the need for statistical models able to associate genotypes from individuals and interacting SNPs to phenotypic traits such as diseases, physiological and productions traits [1]. Our paper is a contribution to this association problem.

The systematic exploration of the universe of variants spanning the entire genome through genome-wide association studies (GWAS) has already allowed the identification of hundreds of genetic variants associated to complex diseases and traits, and provided valuable information into their genetic architecture [1] while allowing to improve prediction of phenotypic outcomes [2]. Nevertheless, most variants identified so far have been found to confer relatively small information about the relationship between changes at the genomic locations and phenotypes because of the lack of reproducibility of many of these findings, or because the identified variants most of the time explain only a small proportion of the underlying genetic variation [3]. This observation, quoted as the ‘missing heritability’ problem [4] of course raises the following question: where does the unexplained genetic variation come from? Several authors have postulated that many genes and mutations could be involved, with individual small effects, resulting into a low detection power in most of the performed studies, but with large collective effects [5]. Another tentative explanation is that genes do not work in isolation, leading to the idea that sets of genes (“gene networks”) could have a major effect on the tested traits while almost no marginal effect is detectable at individual locus level. Note also that this gene network hypothesis is a potentially credible explanation to the lack of reproducibility of obtained positive results [6], due to situations where different mutations or mutations combinations within the network (within the same genes or on different genes in the networks) could lead to similar phenotypic effects [7].

Consequently, an important question still remains about the exact relationship between the genomic configuration, including the interactions between the involved genes, and the phenotypic expression. The major idea in this respect is to try to associate observed variations at the macroscopic level (phenotype) to identified variations and their interactions at the molecular level.

This view introduces at least two challenges. First, the genetic mechanisms underlying most traits of interest are complex and probably involve most of the time many genes and many interactions between these genes, leading to a complex relationship between genomic variants and phenotypes. So, modeling and identification of every, and even of any, interaction is a potentially very challenging task [8]. Second, from a more statistical point of view, fully modeling the interactions leads to models with large number of parameters to be estimated and large search space, leading to the well-known ‘curse of dimensionality’ problem [9]. Furthermore, increasing the number of parameters to be estimated potentially makes the power issues mentioned above even more critical. Nevertheless, introducing interactions into the model might lead to a more accurate model of the underlying genetics, which in turn might improve the detection power of effects of interest. So it is not obvious that interaction models will present poor power when compared to non-interaction ones, which should motivate more research on the subject.

In the literature, various statistical methods have been used to detect gene-gene or gene-environment interactions [10, 11]. Many of these statistical methods are parametric and rely on large samples properties [12, 13]. On the other hand, nonparametric methods have generated intense interest because of their capacity to handle high-dimensional data [14]. In order to limit the size of the search space, many of the proposed approaches may have missed potential interactions by only considering variants that have a significant genetic marginal effect as, for example, in the logistic regression method proposed by [15], where the model relates one or more independent variables (i.e., main effects for genes) and their corresponding interaction terms (i.e., gene-gene interaction effects) to a discrete dependent variable (e.g., disease status). Because of issues linked to the dimensionality, models such as the logistic regression are limited in their ability to deal with interactions involving many factors [16]. In response to these limitations, novel methods for detecting interacting variants have been designed, such as neural networks [17], random jungles [18], random forests [19], BOOST “BOolean Operation-based Screening and Testing” [20], support vector machine [21], MegaSNPHunter [22], AntEpiSeeker [23] or odds ratio [24].

One of the most successfully used family of methods in the gene-interactions problems is multifactor dimensionality reduction (MDR) [16]. The MDR method is nonparametric (i.e., makes no hypothesis about the distribution of the statistical parameters), model-free (i.e., it assumes no particular inheritance model), and directly applicable to case-control and discordant-sib-pair studies [25]. The main idea in MDR is to reduce the dimensionality of multi-locus data to improve the ability to detect genetic combinations that confer disease risk [26]. MDR has been proposed to identify gene–gene or gene-environment interactions when marker and/or environment information is available [26]. An advantage of the MDR methods is, as pointed out in [27], that, due to their nature, they theoretically allow to highlight gene–gene interactions of any order [28].

Refinements of the method have been proposed to deal with potential limitations. Cattaert et al. [29] has proposed a novel multifactor dimensionality reduction method for epistasis detection in small or extended pedigrees, FAM-MDR. Cattaert et al. [30] and [31] have also developed Model-Based Multifactor Dimensionality Reduction (MB-MDR), a MDR-based technique that is able to unify the best of both nonparametric and parametric worlds, allowing to include corrections for cofactors, as in parametric models, while using the flexible framework of non-parametric MDR analyses. Another extension is Generalized MDR (GMDR), a version of the MDR method that permits adjustment for discrete and quantitative covariates and is applicable to both dichotomous and continuous phenotypes [32].

Although applied to numerous genetic studies [33, 34], MDR faces important challenges. First, MDR can be computationally intensive, especially when a large number of markers needs to be tested [26]. Second, the interpretation of MDR results is difficult, for example in situations where a strong marginal effect makes the effects of the other polymorphisms in the interaction questionable [31]. Third, the MDR method can fail in finding the correct models, because it assumes that there is no genetic heterogeneity, as in situations where a group of cases are explained by a combination of loci different from the one that explains another group of cases [30]. Lastly, the number of possible combinations explodes exponentially with the number of interacting factors, which makes the approach impractical in terms of needed cohorts sizes and computing time in situations where large numbers of genetic and/or environmental determinants are involved, another instance of the ‘curse of dimensionality’ problem.

In this paper, we propose a novel MDR approach using K-Nearest Neighbors (KNN) methodology (KNN-MDR) for detecting gene-gene interaction as a possible alternative to current MDR methods in situations where the number of involved determinants is potentially high and the number of tested markers is large. After explaining the rationale of our method, we will provide results on the comparison of KNN-MDR to a set of competitor methods on both simulated and real datasets.

## Methods

### KNN method

KNN stands for “K Nearest Neighbors” and is one of the most popular algorithms for pattern recognition and classification. Roughly, classification of an observation can be made using a majority vote within the K nearest neighbors of the observation [35], where the neighborhood is based on a defined distance between observations. Although simple, many researchers have found that the KNN algorithm accomplishes very good performance in their experiments on different data sets [36]. Also, KNN is a multivariate method that retains the variable relationships seen in the data because the logical relationships among response variables will be maintained [37], a feature of importance in our genetic context. The flexibility of KNN is also a great advantage and this technique helps to alleviate the curse of dimensionality by shrinking the unimportant dimensions of the feature space, bringing more relevant neighbors close to the target point [35].

### MDR method

The method will be described for dichotomous traits for the sake of simplicity, but could be extended to other situations using the approach described for GMDR [32]. The Multi-Dimensional Reduction (MDR) method is designed to replace large dimension problems with reduced dimension ones, allowing to make inferences based on a smaller set of variables. In the context of genomic studies, the idea in [26] is to replace the high dimensional problem arising from considering several markers simultaneously, with one unique variable (for example, a status) that can take only 2 values (for example, ‘*high risk*’ or ‘*low risk*’). To illustrate, if a set of N SNP markers is used in a case-control study to define the multi-locus genotype, 3^N^ genotypes are possible. Each of these genotypes can be mapped to a status with only 2 values (case or control) using a majority vote on the statuses of the training set individuals falling into that genotype. The classification performances of any set of markers used to define the genotypes can then be assessed, typically using a cross-validation procedure, where the performance is estimated on a test set for each partition trough a measure involving sensitivity and/or sensibility of the classifier, and averaged over all partitions. For all computations reported in this paper, we have used a 10-fold cross-validation procedure and assessed the performances using ‘balanced accuracy’, which is a simple average of the sensibility and the sensitivity of the classifier. Repeating this procedure over all possible markers sets allows obtaining the best model, which is defined as the set of markers providing the best allocation performances. In practical situations, the potential number of tested markers sets might be huge: if an exhaustive search is to be performed on all P-markers interactions in a GWAS with M markers, about M!/[P!*(M-P)!] ~ M^P^/P! combinations would need to be checked, a huge number with nowadays available markers panels. Significance for the optimal model can be obtained through a permutations test, in which the potential links between the individuals’ genotypes and the phenotypes are disrupted by randomly shuffling the phenotypes. The *p*-values obtained using this test have then to be corrected for multiple testing, where multiple tests are due to the number of models that are successively tested.

### KNN-MDR method

Although a widely used and well-established technique, MDR faces several problems, as detailed above. The computational load described in the previous section remains a major issue. Although recent publications [38] have provided some tools to achieve low order interactions screening in a GWAS, the task will remain very challenging for larger order interactions and for larger markers sets, such as sequencing data, and alternative approaches reducing the computer burden remain desirable. Another problem linked to the MDR methodology arises when a test set individual’s multi-locus genotype has not been observed in the training set, making it impossible to classify the newcomer. Furthermore, in situations where very few training individuals share the same multi-locus genotype as the tested one, the accuracy of the assignment can also be questioned. Since the number of multi-locus genotypes explodes exponentially when the number of markers in the markers sets increases, this problem becomes rapidly critical, and could finally render the approach inaccurate (few individuals are used to classify) or even unusable (no individual useable to classify) in situations where more than 3–4 markers are to be used simultaneously and with classical cohorts’ sizes. Another consequence of the limited number of markers that can be considered simultaneously in MDR is that the genomic regions involved in interactions will most of the time be represented through a single marker, although, due to linkage disequilibrium, considering several linked markers might increase the association signal intensity, and consequently improve the detection power.

Our proposal is therefore to slightly modify MDR to allow facing some of the shortcomings of the method. The only modification is in the status allocation procedure: while MDR uses a majority vote among the (potentially scarce or empty) set of individuals sharing the same multilocus genotype as the tested individual, we propose to use a majority vote within a set of the K nearest neighbors of the tested individual. This procedure has the obvious advantage to eliminate the problem of potentially scarce or empty genotypic configurations mentioned above. On the other hand, this strategy introduces the need to define the neighborhood: a “distance” between individuals based on the genotypic configurations at the selected markers will be needed, and the size K of the neighborhood will have to be provided. These parameters of the method - the chosen distance, K - are further discussed in the Discussion section. A second advantage of our approach is that more markers can be considered at once than in the classical MDR strategy. The idea, also detailed in the Discussion section, is thus to replace the sets of single markers used in MDR by sets of windows spanning several markers: the M markers are split into W windows of contiguous markers, where the choice of the windows sizes and positions could use genetic criteria explained in the Discussion section, and the distances used in KNN-MDR are based on these windows. All the other steps are similar to the classical MDR steps (partitioning for the cross-validation, performance and significance assessments, best model selection). Note that the number of windows W might be much smaller than the number of markers M, as explained below. Consequently, the proposed approach might greatly reduce the needed amount of computations, and consequently make higher-order interactions more affordable. Although alternatives are possible, we have used Mahalanobis distances in our analyses because of its numerous advantages in our setting (see the Discussion).

Note that, in KNN-MDR, the computer burden scales quadratically with the number of individuals since the distances between pairs of individuals are needed, but is less sensitive to the number of markers since markers are pooled into windows. So, the important parameter from a computing point of view is the number of windows W, which does not necessarily increase when the number of markers increases.

### Competitor methods

After designing our method, we needed to compare the performances of our approach to some of the other proposed algorithms. Since many methods are available [2], we decided to consider four of the most popular ones to be used in the comparison, namely: MDR, BOOST, MegaSNPHunter and AntEpiSeeker. The rationale for choosing this set of methods is the following:

- AntEpiSeeker [23] and BOOST [20] have been recommended as efficient and effective methods in the comparative analysis of [39],
- MDR [26] is one of the most famous methodologies for detecting interactions [2],
- MegaSNPHunter [22] is targeting high level interactions, one of the potential advantage of KNN-MDR. Also, a method for exploiting large genotypes sets is provided, which is another objective of our algorithm,
- All these methods have been applied successfully to real datasets,
- These methods have different search strategies: exhaustive search (MDR, BOOST), stochastic search (MegaSNPHunter) and heuristic search (AntEpiSeeker),
- Software implementing the methods is available.

### Simulation

In order to assess the performances of the proposed method, we have simulated various situations and ran MDR, BOOST, MegaSNPHunter, AntEpiSeeker and KNN-MDR on the same datasets to compare the performances in terms of detection power and accuracy. The generation of the simulation datasets will be described in the following lines.

One of the aims of our study was to assess the performance of the methods to unravel gene-gene or gene-environment interactions in the absence of large marginal effects. The reason for that choice was that many methods are able to detect such large marginal effects and to infer interactions within a limited set of loci selected on that basis. Accordingly, we wanted to devise an approach that is able to detect interactions even in the absence of marginal effects. For that reason, efforts have been devoted to generate datasets with interacting genes in the absence of significant marginal effects. Furthermore, heterogeneity between samples has been shown to be a major source for the non-reproducibility of significant signals [40]. We have modeled heterogeneity by associating penetrances to the multi-locus genotypes underlying the simulated binary trait. The data generation algorithm proceeds along the following lines:

1. To obtain a linkage disequilibrium (LD) pattern similar to patterns that can be observed in humans, SNPs spanning the human chromosome 9 (HSA9) have been obtained from a study on Crohn disease in Caucasians [41] for 197 individuals. Two thousand markers with minor allele frequencies (MAF) above 0.3, and no missing genotype have been selected. Hardy-Weinberg equilibrium tests have been performed on the genotypes for these markers, and the high MAF threshold has been chosen to select informative markers among the complete list of markers, to compensate for the information loss resulting from discarding the other available markers to decrease the computational load. Nevertheless, since experimental data has been used, genotyping errors might be present. Presence of LD in the data was checked using simple association tests between consecutive markers (data not shown).
2. Since many different individuals are needed in the simulations, we used a trick similar to [42] to generate new individuals based on the few available genotypes: each individual genotype was chopped into 10 SNP windows, leading to 200 windows with (maximum) 197 different 10 loci genotypes. Each simulated individual genotype was then built by randomly sampling a genotype for each window and concatenating the 200 genotypes into a new complete genotype with 2000 markers. This technique allows for 197^200^ potentially different individuals while conserving some LD.
3. G SNP were then randomly chosen as having an effect on the simulated phenotype, where G = 2, 3, 4 or 5. Since SNP selection is random, SNP might be linked or not.
4. Selected SNP genotypes were then used to generate the binary phenotypes. More details of the algorithm are given in an appendix (see Additional file 1), but roughly:
  - A penetrance is computed for each multi-locus (G SNP) genotype in such a way that each of the G SNP shows no marginal effect:
    $$ \mathrm{P}\left(\mathrm{A}\ \Big|\ {\mathrm{G}}_{\mathrm{i}} = 0\right) = \mathrm{P}\left(\mathrm{A}\ \Big|\ {\mathrm{G}}_{\mathrm{i}} = 1\right) = \mathrm{P}\left(\mathrm{A}\ \Big|\ {\mathrm{G}}_{\mathrm{i}} = 2\right) = \mathrm{P} $$
    where G_i_ denotes the genotype for locus i (i = 1, 2, …, G), 0, 1, 2 are the number of instances of the minor allele in the SNP genotype, A means Affected, P(A | G_i_) is the penetrance for genotype G_i_, and P is the prevalence of the disease in the sample (since we used a more or less balanced case-control design, we used a prevalence of *P* = 0.5).
  - The multi-locus penetrances MP = P(A | G_1_ = k, G_2_ = m, …) where k, m, … = 0, 1 or 2 are obtained to meet the requirement of no marginal effect (see previous step). An algorithm to compute these penetrances is provided in an appendix (Additional file 1).
  - The phenotypes (i.e., affected or non-affected status) are then obtained by randomly sampling a uniform distribution between 0 and 1 and comparing the obtained deviate d to the multi-locus penetrance MP: if d < (>) MP, the individual is (not) affected.
5. One SNP out of 2 consecutive SNPs was then randomly discarded, leaving 1000 markers for the analyses. The rationale of this selection is that causative mutations might nowadays be present or not in the genotyped variants. This will also be the case in our simulations.
6. Genotypes and corresponding phenotypes were generated for each simulation, and the obtained datasets were studied using all four methods. KNN-MDR windows size was set to 10 markers, leading to 100 non-overlapping windows, and K value was set to 10. The parameters for the other methods were chosen so that resolution was almost similar for all methods.
7. Finally, 100 permutations of the phenotypes were performed for each simulation (unless otherwise stated) and the resulting datasets were analyzed using the four methods in order to assess significance. Although this number of permutations is too low for routine work, it was used to reduce the computing burden and help us to discriminate between results clearly non-significant (i.e., *p* > 0.05) and those potentially significant (i.e., *p* < 0.05). When a higher precision was needed for the *p*-values (see below), an adaptative permutations scheme was used, in which windows not reaching a pre-determined *p*-value threshold are progressively abandoned in the permutations scheme since these windows are very unlikely to finally reach a significant result [43].

### Real data

Analyses using real data have also been performed. Rheumatoid arthritis (RA) genotype data on 1999 cases and 1504 controls have been obtained from WTCCC [44]. Genotypes from the Affymetrix GeneChip 500 K Mapping Array Set have been filtered using the usual quality controls tests on DNA quality (percentage of genotyped marker for any given individual above 90%), markers quality (percentage of genotyped individuals for any given marker above 90%), genotypes frequencies (markers with a *p*-value below a Bonferroni adjusted 5% threshold under the hypothesis of Hardy-Weinberg equilibrium in the controls cohort have been discarded). Missing genotypes for the GeneChip markers have been imputed using impute2 software [45]. This procedure led to 312583 SNP to be analyzed for the 2 cohorts. Zhang et al. [46] and [47] also used this dataset to infer potential interactions. These studies will therefore serve as a comparison for the results obtained with our approach.

### Working on large datasets

When working on large sets of markers, such as for example those commonly met in GWAS analyses, splitting the complete set into a reasonable set of windows could necessitate including large numbers of markers in each window, which would eventually swamp the signals of interest, as explained in the Discussion section. An alternative is to pre-select a subset of markers (for example, taking one marker every N markers) and to define a first set of windows based on these markers. This strategy would allow windows to cover potentially large regions while preserving some detection power. After a first run of KNN-MDR using this subset, the detected regions (i.e., those departing significantly from the distribution of the results, assuming that most combinations do not have an effect on the studied trait, and that this distribution accordingly corresponds to the distribution of the used measure under the null hypothesis) would be used for a second round of KNN-MDR runs. In this new round, the markers hidden in the first round could be partially or totally recovered for each of the identified regions, and the same approach as in the first round could be used recursively on these new regions. The sequential detection of progressively denser regions could continue down to single markers. An example of this strategy in a GWAS study is provided in the of “Results on WTCCC data” section.

## Results

### Results on simulated data

Since performing classical MDR analyses on a large number of markers is not an obvious task, especially when the number of putative involved SNPs (noted G) is 3 or more, we restricted our analyses to G = 2 and G = 3 to make comparisons to other methods feasible. We have defined the “power” as the proportion of simulations where an association signal was detected (*p* < 0.05), and the “corrected power” as the proportion of simulations where the association was detected and involved the causal SNP (i.e., a rough measure of accuracy). The comparison of the five tested methods is presented for situations where G = 2 in Table 1 and for G = 3 in Table 2 (data sets used to generate these 2 tables are provided as Additional files 2 and 3 and more details on the comparisons of the methods results are provided in Additional file 4).

**Table 1:** Simulation results when G = 2 and the number of cases and controls is 500

Method	MDR	AntEpiSeeker	BOOST	MegaSNPHunter	KNN-MDR
Power	0.68	0.88	0.76	0.84	0.81
Corrected power	0.56	0.39	0.48	0.20	0.71

**Table 2:** Simulation results when G = 3 and the number of cases and controls is 500

Method	MDR	AntEpiSeeker	BOOST	MegaSNPHunter	KNN-MDR
Power	N/A	0.65	0.67	0.80	0.74
Corrected power	N/A	0.15	0.28	0.12	0.63

Tables 1 and 2 shows the results of 100 simulations. For KNN-MDR, the number of neighbors is set to 10, and the 1000 markers are split into 100 windows of 10 consecutive markers. All possible sets of up to 2 windows for Table 1 (5050 sets) and up to 3 windows for Table 2 (166750 sets) have been tested. Parameters for the other methods were set to default values. Due to the very large number of tests required when 3 markers are involved, MDR results have not been obtained in Table 2. Data sets used to generate these 2 tables are provided as Additional files 2 and 3

As can be seen from Tables 1 and 2, KNN-MDR seems to show reasonable power when compared to its competitors. More importantly, corrected power of the method is significantly better than for the other tested methods (after 100 simulations, *p* = 0.0143 when comparing KNN-MDR to its closest competitor for G = 2 and p = 7.23e-7 for G = 3).

A short literature survey [2, 48–50] leads to the conclusions that many of the methods seem to be marred by high false positive rates. To test that, we have simulated situations where no SNP was involved in the generation of the phenotypes, so that SNP detection by the algorithms would correspond to false positives. Table 3 shows the results of these simulations.

**Table 3:** Simulation results when G = 0 and the number of cases and controls is 500

Method	MDR	AntEpiSeeker	BOOST	MegaSNPHunter	KNN-MDR
Power (*p*-value <0.05)	0.18	0.45	0.19	0.38	0.07

The detection threshold α is set to 0.05. The data set used to generate this table is provided as Additional file 5

We ran another set of simulations to assess the respective effects of the sizes of the windows and of the number K of neighbors on the (corrected) detection power. Results of these simulations are reported in Table 4.

**Table 4:** Power (above) and corrected power (below) when the parameters K (number of markers) and W (windows size) are varied in 100 simulations with 500 cases and 500 controls and G = 2

		W=			
		5	10	15	20
K=	5	71	68	62	52
		65	62	51	38
	10	70	66	64	56
		60	53	51	43
	15	71	65	59	58
		59	49	47	44
	20	69	60	56	53
		67	55	52	45

The data set used to generate this table is provided as Additional file 6

### Results on WTCCC data

Since working on such a large dataset (>300 k SNP) is very demanding in terms of computing time, we proceeded as follows:

1. 20 k SNP were first extracted from the data. Although several selection procedures could be applied, we simply selected 1 SNP every 15 SNP.
2. We divided the data into 200 windows of 100 SNP each.
3. We then tested each of the 19900 possible pairs of windows (sets of 200 SNP) using KNN-MDR.
4. We extracted the 83 sets for which the *p*-values were lower than 2.5e-6 (a threshold obtained after Bonferroni correction at level 0.05). To reach that significance level using a permutations procedure, we used the following adaptative scheme: after 100 permutations performed on the 19900 possible pairs of windows, only those reaching the 0.05 level were considered for the next round of permutations, assuming that those not reaching that level of significance were very unlikely to reach the desired significance at the end of the process. This left us with 2319 combinations. In the next round, 900 more permutations were performed, and only the combinations reaching the 0.005 level were kept (i.e., 1207 combinations). Repeating this procedure for 1.0e4, 1.0e5, 1.0e6 and 2.0e6 permutations, and respective thresholds equal to 5.0e-4, 5.0e-5, 5.0e-6 and 2.5e-6, we ended up with the 83 sets cited above.
5. The SNP hidden in step 1 were then recovered, leading to 83 sets of 3000 SNP (i.e., 200*15).
6. KNN-MDR was applied on every set from step 5: the sets were divided into 30 windows of 100 SNP and all 435 combinations of windows pairs in each set were considered by KNN-MDR.
7. We kept the 241 sets of 200 SNP with a *p*-value < 1.15e-4 (Bonferroni correction at level 0.05).
8. MDR was then used for the sets from the previous step, leading to examine 19900 SNP-SNP interactions for each set.
9. The interactions with a *p*-value < 2.51e-6 (Bonferroni correction at level 0.05) were then considered as significant.

**Fig. 1:**
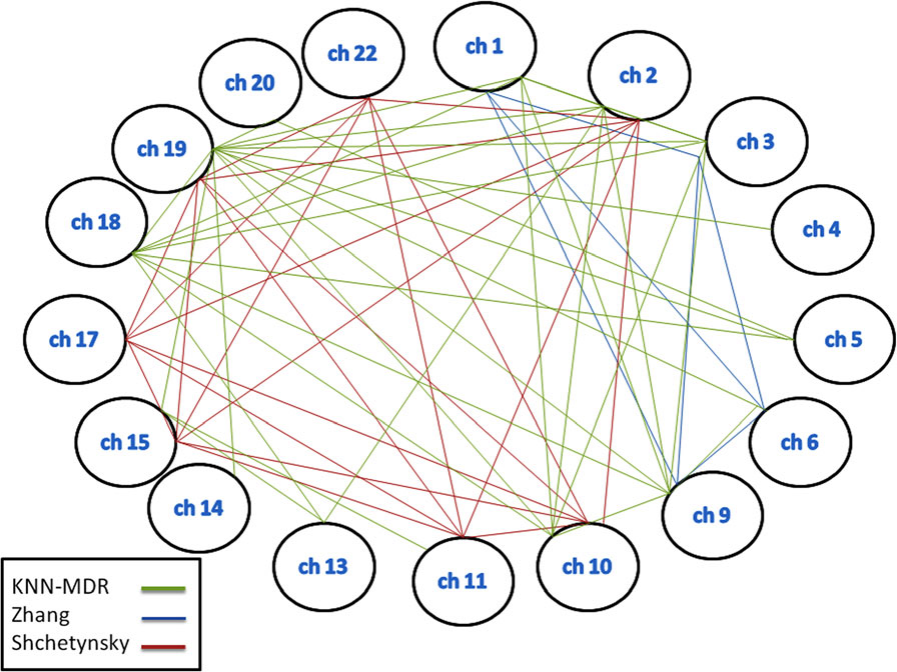
Comparison of the inter-chromosomal interactions detected on the RA dataset by KNN-MDR and other interaction methods using this same dataset as example (Shchetynsky et al. [47]; Zhang et al. [46])

Results from this analysis are presented in Table 5. The full version of Table 5 is provided in a supplementary file. Figure 1 provides a view of the significant results at the chromosome level for our study as well as for 2 other similar studies on this dataset ([46] and [47]).

**Table 5:** The 10 most significant results of the analysis on the RA dataset from WTCCC

SNP	Position	Testing balanced accuracy	*P*-value
rs10979420, rs778980	9:108634242, 19:5863725	0.89	2.51*10-6
rs10979420, rs778982	9:108634242, 19:5866574	0.89	2.51*10-6
rs6781338, rs778982	3:180060018, 19:5866574	0.89	2.51*10-6
rs778980, rs17325560	19:5863725, 20:2614933	0.89	2.51*10-6
rs4979291, rs10979420	9:107732763, 9:108634242	0.89	2.51*10-6
rs561259, rs10979420	2:79014325, 9:108634242	0.89	2.51*10-6
rs1862333, rs17325560	5:181066946, 20:2614933	0.89	2.51*10-6
rs1862333, rs485409	5:181066946,18:28918712	0.89	2.51*10-6
rs571307, rs578044	13:29942173,18:28918696	0.89	2.51*10-6
rs1169565, rs571307	2:71196518, 13:29942173	0.88	2.51*10-6

The first two columns provide the names and chromosomal positions of the SNP found to be associated to the phenotype. Positions are indicated by the chromosome and the SNP physical position on the chromosome using the NCBI human build 35. The third column contains the corresponding balanced accuracies and the last column reports the *P*-values computed using an adaptative permutation scheme. The complete table is provided as Additional file 7

## Discussion

This paper has introduced a new MDR approach to find markers interactions in genomic scans. It could also be used for other attributes than markers, such as environmental factors, leading to a gene-environment interaction search method. Due to the proposed strategy relying on the MDR approach, and in parallel with a recent study [42] using (“simple”) MDR as a reference strategy, we have compared our proposed method’s performances to this reference and other reference methods (MegaSNPHunter, AntEpiSeeker, BOOST), and tried to show that our method could have benefits compared to these methods. Of course, other algorithms might have been tested, such as the recent Bayesian High-order Interaction Toolkit [51] which is proposing a MCMC approach to scan the very large search space of potential sets of markers (incidentally, this algorithm has also been tested on a smaller set of simulations, and its power has been found significantly lower than KNN-MDR on this dataset). Our point in this respect was not to be exhaustive, but simply to show that the approach we propose can bring some more information than other popular methods, and might be a useful addition to the arsenal developed to tackle genomic interaction problems.

The results obtained through the simulations demonstrate some of the features that potentially make KNN-MDR helpful. More specifically, the simulations show the feasibility of scans using large number of markers, as opposed to MDR where the computer burden explodes with the number of markers (when it simply increases linearly with KNN-MDR). This might allow to highlight interactions between markers far apart on the genomic map (trans-interactions), while some strategies proposed to restrict the scans to close-by markers (cis-interactions) to reduce the amount of computations.

We now discuss some of the features of the method:

### Number of interacting loci

In this paper, although the algorithm given in the appendix can be used for G larger than 3, only 3 markers have been used to generate the phenotypes. Nevertheless, in practical applications, it is not unlikely that situations involving more than 3 loci might exist. These situations might increase the interest of using methods such as KNN-MDR. Indeed, when more regions are involved in the phenotype, this could decrease the distance measure between individuals sharing some or all of these regions and better cluster individuals sharing the same status. Conversely, in MDR, discovering such complex patterns would likely necessitate to increase the number of loci scanned simultaneously, which would make computations even more difficult. Also, increasing the number of loci increases the number of cells with no (or very few) observations, making status allocation potentially inaccurate or even impossible.

### Parameter settings

We mentioned earlier that parameters setting in KNN-MDR mainly involves defining the sizes, positions and the number of windows, the number K of neighbors and the distance measure. All parameters are problem dependent, making it difficult to devise general rules. Nevertheless, some guidelines might be given.

In all the analyses performed in this study, we have only used Mahalanobis distances, as already mentioned. The reason was that this distance allows to take into account potential correlations between attributes (typically, linkage disequilibrium between close markers) and because it makes it possible to weight the attributes in the sum (for example to take into account that similarity for rare alleles is more informative that on frequent ones). In our studies, only SNPs have been used, for which the distance proposed in the Mahalanobis measure makes sense, with D(AA, AB) = D(AB, BB) = 0.5* D(AA, BB), where AA, AB and BB are the three possible SNP genotypes. This might be different and might need more investigations if other types of genetic variants are used. Note also that, in most computations, to reduce the computational burden, the correlation between neighboring markers has not been estimated but set to 0 (i.e., we used the normalized Euclidean distance), which might potentially affect the power. Although we did not explicitly test this, we expect that including the correlations would lead to better take into account the linkage disequilibrium, which should have a positive effect on the detection power. So, using this information might be favorable in terms of power, but at the cost of an increase in the computation time. Note also that using this kind of distance makes less sense when working with markers with more than 2 alleles, unless it can be postulated that the distance between, for example, alleles 1 and 3 is roughly twice the distance between alleles 1 and 2. An easy to compute and similar distance measure would then be to square the number of differing alleles (0, 1 or 2) between two compared genotypes, to normalize as for the Mahalanobis distance, to sum over all markers in the window and to take the square root of the product. This “binary” distance is implemented in our KNN-MDR software.

For the windows dimensions, our idea is to use the assumption that individuals sharing mutations responsible for the trait should look more similar in the surroundings of these mutations than those not sharing these mutations. The resemblance should thus extend to neighboring markers, where the neighborhood size is a function of the linkage disequilibrium (LD) in the region. In situations where LD increases (due to the studied population and/or the markers density), distance between individuals sharing genomic regions (including the causal regions) should decrease and detection power should increase. Note that this genomic feature is ignored in the other tested methods. Accordingly, the windows sizes W should ideally be defined to capture the local linkage disequilibrium. Since the measurable LD is dependent on the population history and on the markers density, assessment of this measure should first be made in order to have reference dimensions for the various windows to be used in KNN. Note that the extent of LD need not be the same across the whole genome: accordingly, the size of the windows might be varied along the genome to better reflect the underlying structure and better capture the relevant information.

To illustrate that expected behavior, we have performed the simulations leading to Table 4. As visible from that table, the powers decrease when the windows sizes increase. Our interpretation of this result is that, due to the way the simulated data are generated, chunks of five linked (i.e., showing some LD) markers are used, which should restrict the signal caused by LD to five markers. Adding more markers to the windows adds noise, and consequently reduces the resemblance between the composite pieces of chromosomes harboring the causative mutations, and thus the power.

Next, the number K of neighbors should somehow reflect the number of animals sharing regions harboring causal mutations. This number is of course unknown and difficult to evaluate a priori because it is dependent on various population and trait parameters such as the history of the population or the genetic heterogeneity of the trait. Furthermore, it might vary from region to region, making it difficult to devise general rules allowing to infer relevant values of K. Possible “brute force” approaches would be to rerun the algorithm with varying number of neighbors (grid search) or to use bootstrap methods [52]. This strategy could allow to capture regions of interest while integrating potential sources of variations, at the cost of supplementary computer burden. Another point of view is that the corrected powers do not significantly (at the 5% level) disagree between the various K values for the tested windows sizes, which indicates that the results might not be very sensitive to this parameter, at least in our simulations. For this reason, we used K = 5 or K = 10 in our computations. Note also that odd K values might facilitate the majority vote.

### False positive rates

Our simulations have shown that, as reported in other studies, results are often penalized by high false positive rates (Table 3). One obvious reason is multiple testing: the large number of performed tests necessitates that the significance threshold be properly adapted, which is not always easy to do. Another reason in our study is the way we have performed the simulations. Indeed, we have managed to have epistatic interactions with little marginal effects in order to avoid the easier situations where individual loci can be identified in a first step, followed by the identification of interactions between these loci identified first in a second step. To obtain these situations, we have used multi-locus prevalences, which has led to some kind of genetic heterogeneity: a same multi-locus genotype could simultaneously be present in cases and in controls, making it harder to identify these loci. These complicating factors have been associated to higher false positive rates in other studies, along with other design factors such as the number of cross-validation subsets [30, 49, 53]. Our model might be less sensitive to these factors: looking for neighbors might allow selecting the individuals sharing the relevant features in a heterogeneous set of individuals. Also, decreasing the number of tests (in comparison to MDR, for example), might also lead to somehow relaxing the penalty arising from multiple testing.

### Power and corrected power

The reason for the drop in the power of the alternative methods when considering the accuracy is not completely clear, but we can suggest a tentative explanation.

As can be seen from Table 3, all methods show high rates of false positive results, while KNN-MDR seems to behave reasonably well from that point of view. Although this is no definite proof, this is an indication that the high power observed in the simulations for the alternative methods is probably due to false positive results. Correcting for the accuracy (using “corrected power”) therefore eliminates most of these false positive results, so drastically reducing the observed power.

A potential criticism on our “accuracy measure” is that using windows sets makes it more likely to cover the culprit regions, and so this “accuracy” measure is biased in favor of KNN-MDR. For that reason, and to make the comparison fair between the methods, we have chosen the parameters to end up with similar number of markers in the finally selected markers sets in each approach. Note nevertheless that the resolution of KNN-MDR could eventually be increased in these analyses, for example using the strategy described for large datasets in the Methods section.

Figure 1 shows that no combination at the chromosome level is consistent across our study and two other similar studies on the same dataset ([46] and [47]) while other significant results are specific to one or two methods. Some results from KNN-MDR are consistent with those obtained by Shchetynsky, others are consistent with those of Zhang while no corresponding results between Zhang and Shchetynsky studies could be found. Power and false positive issues might potentially explain these discrepancies, although no definite proof can be put forward based on these preliminary analyses.

So, in our study as in the other ones, statistically significant SNP interactions have been identified using KNN-MDR and MDR in a genome-wide association study. Their biological relevance is obviously not clear at this stage and needs more investigations in the future. We can nevertheless say that some of our results are consistent with other results in the domain of Rheumatoid Arthritis ([20, 46, 54]) and that, in addition, new candidates contributing to the etiology of this disease have potentially been identified. This result shows that, as suggested in the simulations, differences in the approaches and potential differences in the respective powers of the used methods might lead to new insights in the etiology of the disease. This observation should trigger more research on the use of composite methods, combining the qualities of several approaches.

### Computer resources

In our results, the comparisons between (MegaSNPHunter, AntEpiSeeker, BOOST, MDR) and KNN-MDR in terms of computer resources has not been fully addressed. Nevertheless, it has been shown how and why KNN-MDR decreases the computer load with respect to MDR, making it a potential candidate to analyze large datasets, as shown for the RA data. To be fair, it should be mentioned that computing nearest neighbors is more computer intensive than a majority vote in the subset sharing the same multi-locus genotype. Nevertheless, as shown in the simulations, and as can be understood from the previous discussion, computations remain more affordable in KNN-MDR than in MDR and the other methods for similar scans. Furthermore, strategies could also be devised to make KNN-MDR efficient, such as pre-computing distances for windows and using distance additivity properties to compute distance over several windows.

Another point that might be worth adding is that, although KNN is natively a classification method, we have used it here in a detection context. KNN-MDR could nevertheless as well be used as a classification tool: to that end, the best model (i.e., the best set of markers) could be used to compute the neighborhood of a new individual and classify the latter in one or the other category.

## Conclusions

In summary, KNN-MDR is an alternative to existing methods for detecting epistatic interactions, with interesting features. Among these, we have demonstrated that KNN-MDR is more computationally efficient than other exhaustive strategies, facilitating the analysis of large-scale data sets with potentially genome-wide SNPs. The method is also capable to detect high-order interactions and to take into account linkage disequilibrium (LD). Another advantage is that it is able to detect interactions between SNPs even in the absence of marginal effects. Also, the method is non-parametric: no prior distribution is assumed, unlike many parametric-statistical methods. Nevertheless, parameters (distances, number of neighbors, windows definition) are available to allow some flexibility in the search strategies, which could help to render the method useful in other classification contexts.

Although KNN-MDR is potentially beneficial for epistasis detection, several aspects would nevertheless deserve more investigations. For example, the burden associated to the computation of the K nearest neighbors could become an issue when the dataset is very large. Since the load increases quadratically with the number of individuals, and linearly with the number of markers, improving the computational performances of the method could necessitate some code optimization to make the program more efficient. Another point necessitating more work is the tuning of the parameters allowing an optimal detection power. This includes the optimal sizes of the windows - which should be dependent on the studied population, the markers density, the LD pattern, the optimal size of the neighborhoods to be considered, the pre-selection of markers in the early phase of large dataset analyses, the distance measure or the adaptative selection scheme for the selection of markers in large studies, among others.

## Additional files

Additional file 1: Computing multi-locus penetrances. (DOCX 19 kb)

Additional file 2: The data set(s) supporting the results of Table 1. (ZIP 22742 kb)

Additional file 3: The data set(s) supporting the results of Table 2. (ZIP 28603 kb)

Additional file 4: Competitor methods. (DOCX 14 kb)

Additional file 5: The data set(s) supporting the results of Table 3. (ZIP 7092 kb)

Additional file 6: The data set(s) supporting the results of Table 4. (ZIP 7095 kb)

Additional file 7: Table 5 complete. (TXT 161 kb)

Additional file 8: KNN MDR user’s guide. (PDF 116 kb)

## Abbreviations

BHIT: Bayesian high-order interaction toolkit
BOOST: Boolean operation-based screening
FAM-MDR: Flexible family-based multifactor dimensionality reduction
GMDR: Generalized multifactor dimensionality reduction
GWAS: Genome-wide association study
HSA9: Human chromosome 9
KNN: K-nearest neighbors
LD: Linkage disequilibrium
MAF: Minor allele frequency
MB-MDR: Model-based multifactor dimensionality reduction
MDR: Multi dimensional reduction
SNP: Single-nucleotide polymorphism
WTCCC: Wellcome trust case control consortium
