# Supplementary material for: KNN-MDR: a learning approach for improving interactions mapping performances in genome wide association studies
Source: BMC Bioinformatics. 2017 Mar 21;18:184. doi: 10.1186/s12859-017-1599-7 (PMC5361736; doi:10.1186/s12859-017-1599-7)
Supplement: Supplementary file 1 — Computing multi-locus penetrances. (DOCX 19 kb) [file 12859_2017_1599_MOESM1_ESM.docx]

# Appendix: computing multilocus penetrances leading to the absence of marginal effects

This document describes the procedure when 2 interacting markers are considered. It can be extended to situations where more than 2 markers interact. We will use the following notations:

- $P=p\left( D \right)$ is the disease prevalence,
- $\left( M_{1}=i,M_{2}=j \right)$ denotes a “multilocus genotype” where the first locus (noted $M_{1}$) has genotype i and the second locus (noted $M_{2}$) has genotype j,
- $f\left( i,j \right)$ is the frequency of the multilocus genotype $\left( M_{1}=i,M_{2}=j \right)$,
- $p\left( i,j \right)=p\left( D\left| \left( M_{1}=i,M_{2}=j \right) \right. \right)$ is the “multilocus penetrance”, which is the probability that an individual carrying the multilocus genotype $\left( M_{1}=i,M_{2}=j \right)$ be affected.

An objective in the simulations is to choose the multi-locus penetrances to obtain no marginal effect at either locus:

$p\left( D|M_{1}=i \right)=p\left( D|M_{2}=j \right)=p\left( D \right)$ for all i and j.

To that end, we can write:

$$\begin{matrix} P & = & p(D) \\ & = & \sum_{i} \sum_{j} p\left( D,M_{1}=i,M_{2}=j \right) \\ & = & \sum_{i} \sum_{j} p\left( D|M_{1}=i,M_{2}=j \right)*p\left( M_{1}=i,M_{2}=j \right) \end{matrix}$$

$$\begin{matrix} \Rightarrow& P & \begin{matrix} = & \sum_{i} \sum_{j} p\left( i,j \right)*f\left( i,j \right) \end{matrix} \end{matrix}$$

In this expression, the $p\left( i,j \right)$ are the unknown penetrances and the $f\left( i,j \right)$ are known constants, dependent on the 2 loci involved in the interaction. The penetrance of any genotype can then be expressed as a function of the other genotypes frequencies and penetrances as:

$$p\left( i,j \right)=\frac{\left( P-\sum_{i'} \sum_{j'} p\left( i',j' \right)*f\left( i',j' \right) \right)}{f\left( i,j \right)}$$

where the double sum corresponds to the double sum given above, but excluding the multilocus genotype $\left( i,j \right)$. It is assumed that $f\left( i,j \right)$ is different from 0 (when $f\left( i,j \right)$= 0, no prevalence needs to be computed for that genotype). Note that:

- The maximum value of $p\left( i,j \right)$ (written$p_{M}\left( i,j \right)$) is obtained when all other penetrances are equal to their minimal value (written $p_{m}\left( i,j \right)$):

$$p_{M}\left( i,j \right)=\frac{\left( P-\sum_{i'} \sum_{j'} p_{m}\left( i',j' \right)*f\left( i',j' \right) \right)}{f\left( i,j \right)}$$

In the absence of other constraints, these minimal values are equal to 0, which leads to a maximum value of:

$p_{M}\left( i,j \right)=\frac{P}{f\left( i,j \right)}$.

When this value is larger than 1, the maximum penetrance is 1.

- The minimum value of $p\left( i,j \right)$ (written$p_{m}\left( i,j \right)$) is obtained when all other penetrances are equal to their maximal value (written$p_{M}\left( i,j \right)$):

$$p_{m}\left( i,j \right)=\frac{\left( P-\sum_{i'} \sum_{j'} p_{M}\left( i',j' \right)*f\left( i',j' \right) \right)}{f\left( i,j \right)}$$

 In the absence of other constraints, these maximal values are equal to 1, which leads to a minimum value of:

$$p_{m}\left( i,j \right)=\frac{\left( P-\sum_{i^{'}} \sum_{j^{'}} f\left( i^{'},j^{'} \right) \right)}{f\left( i,j \right)}=\frac{\left( P-\left( 1-f\left( i,j \right) \right) \right)}{f\left( i,j \right)}$$

$=1-\frac{\left( 1-P \right)}{f\left( i,j \right)}$.

When this value is lower than 0, the minimum penetrance is 0.

**Example**: assume a prevalence of 0.20 and the following frequencies configuration:

| A\B | 0 | 1 | 2 | Total |
| --- | --- | --- | --- | --- |
| 0 | 0.04 | 0.32 | 0.14 | 0.50 |
| 1 | 0.03 | 0.15 | 0.12 | 0.30 |
| 2 | 0.03 | 0.13 | 0.04 | 0.20 |
| Total | 0.10 | 0.60 | 0.30 | 1.00 |

--------------------------------------------------------------------------------------------------------

The algorithm to obtain the multilocus penetrances leading to the absence of marginal effects is based on this last formula. It proceeds as follows:

1. Set a range of allowable values for each genotype penetrance. This can be done using the formula above.

**Example (continued)**: this leads to the following tables of minimal and maximal prevalences:

| p_m_ | 0 | 1 | 2 |
| --- | --- | --- | --- |
| 0 | 0.000 | 0.000 | 0.000 |
| 1 | 0.000 | 0.000 | 0.000 |
| 2 | 0.000 | 0.000 | 0.000 |

| p_M_ | 0 | 1 | 2 |
| --- | --- | --- | --- |
| 0 | 1.000 | 0.625 | 1.000 |
| 1 | 1.000 | 1.000 | 1.000 |
| 2 | 1.000 | 1.000 | 1.000 |

--------------------------------------------------------------------------------------------------------

1. Next, the algorithm iterates over each genotype, considering first the ones with the smallest allowable penetrance range. A penetrance value is then randomly chosen for the selected genotype, and the minimal and maximal penetrance for that genotype is set to that value. The range for the remaining genotypes are then recomputed using the minimal and maximal values as described above.

**Example (continued)**: the smallest range is for genotype $\left( 0,1 \right)$. Assume a value of 0.5 has been “randomly” chosen for that penetrance. If we want to compute, for example, $p_{M}\left( 0,0 \right)$, the formula becomes:

$p_{M}\left( 0,0 \right)=\frac{\left( P-p\left( 0,1 \right)*f\left( 0,1 \right) \right)}{f\left( 0,0 \right)}=\frac{\left( 0.2-0.5*0.32 \right)}{0.04}=1$

and for $p_{m}\left( 0,0 \right)$, the formula becomes:

$$p_{m}\left( 0,0 \right)=\frac{\left( P-\left( 1-f\left( 0,0 \right)-p\left( 0,1 \right)*f\left( 0,1 \right) \right) \right)}{f\left( 0,0 \right)}=\frac{\left( 0.2-1+0.04+0.5*0.32 \right)}{0.04}=-15$$

Consequently, the range of the allowable penetrances for this genotype is left to [0,1]. The ranges for the other genotypes are computed similarly, leading to:

| p_m_ | 0 | 1 | 2 |
| --- | --- | --- | --- |
| 0 | 0.000 | 0.500 | 0.000 |
| 1 | 0.000 | 0.000 | 0.000 |
| 2 | 0.000 | 0.000 | 0.000 |

| p_M_ | 0 | 1 | 2 |
| --- | --- | --- | --- |
| 0 | 1.000 | 0.500 | 0.286 |
| 1 | 1.000 | 0.267 | 0.333 |
| 2 | 1.000 | 0.308 | 1.000 |

Based on this table, the next penetrance to be set is for genotype (1,1).

--------------------------------------------------------------------------------------------------------

The algorithm ends after all genotypes have been considered and all penetrances have been obtained. If the procedure fails (because no allowable value remains for one or several genotypes when using the sampled penetrance values for the preceding genotypes in the algorithm), the procedure can be restarted to obtain new penetrance values until a complete set of multilocus penetrances have been obtained.
