# Supplementary material for: KNN-MDR: a learning approach for improving interactions mapping performances in genome wide association studies
Source: BMC Bioinformatics. 2017 Mar 21;18:184. doi: 10.1186/s12859-017-1599-7 (PMC5361736; doi:10.1186/s12859-017-1599-7)
Supplement: Supplementary file 4 — Competitor methods. (DOCX 14 kb) [file 12859_2017_1599_MOESM4_ESM.docx]

# A short description of the use of each method

In our study, the parameters have generally been set to their default values when using the various methods.

Here we explain how the results are obtained in every method.

**KNN-MDR**

The best combinations, containing (10 or 20 or 30) SNPs, are selected as the solution.

The raw power corresponds to the power obtained when the best combination(s) is (are) significant, regardless of if it (they) contains or not the causal SNPs.

The corrected power is the power obtained when the best combination is significant and contains the causal SNPs.

**MegaSNPHunter**

There are four main parameters in the models, including the depth of trees, the threshold for selecting SNPs from trees, the subgenome size and the overlap between subgenome.

1. The depth of trees indicates the depth of SNP interaction. Since most significant interactions are depth 2, so as long as the depth of trees is above 2, the results would not be changed. MegaSNPHunter uses 5 as default setting.

2. The size of subgenome depends on the density of SNP data. Each subgenome should cover the genomic area of possible haplotype effects in practical. Before we start the experiment, we collect some statistics on how many SNPs are genotyped for one gene. This number will be used as the size of subgenome.

3. The overlap between subgenomes is used to solve the boundary problem between genes. Half of the size of subgenome is the best choice. Both the size of subgenome and the overlap between subgenomes depend on the priori knowledge on epistatic interactions.

4. The threshold for selecting SNPs from trees is a very critical parameter to the method.

MegaSNPHunter could rank the importance of SNPs in each subgenome. A cut-off threshold can be used to choose the top ones. The selected SNPs from all subgenomes will first merge together and then compete with each other in the same way at the next level. By having all SNPs compete with each other in training classifiers, MegaSNPHunter reduces the large number of relevant SNPs into a very small set.

The small set contains between 10 and 40 SNPs.

The raw power corresponds to the power obtained when this small set is significant, regardless of if it contains or not the causal SNPs.

The corrected power is the power obtained when the small set is significant and contains the causal SNPs.

**AntEpiSeeker**

In AntEpiSeeker, a two-stage design of ACO (Ant Colony Optimization) is proposed. The first stage of AntEpiSeeker searches SNP sets of sufficient size (larger than the number of SNPs in a given epistatic interaction) using the above ACO, which results in a pre-defined number of highly suspected SNP sets determined by χ2 scores, and another SNP set of a pre-defined size, determined by pheromone levels. The second stage of AntEpiSeeker conducts exhaustive search of epistatic interactions within the highly suspected SNP sets, and within the reduced set of SNPs with top ranking pheromone levels.

For our comparison, we took suspected SNP sets containing more or less 30 SNPs.

The raw power corresponds to the power obtained when this suspected set is significant, regardless of if it contains or not the causal SNPs.

The corrected power is the power obtained when the suspected set is significant and contains the causal SNPs.

**BOOST**

This method examines all two-locus interactions in a screening stage and the ones over a user-specified threshold are then tested in the testing stage.

In the testing stage, two statistic tests, i.e., likelihood ratio test and chi-squared test are conducted to determine whether the interactive effect of a SNP pair is significant.

For our comparison, we took the first 20 SNPs pair, leading to more or less 30 different SNPs.

The raw power corresponds to the power obtained when this SNP set is significant, regardless of if it contains or not the causal SNPs.

The corrected power is the power obtained when the SNP set is significant and contains the causal SNPs.
