# Supplementary material for: KNN-MDR: a learning approach for improving interactions mapping performances in genome wide association studies
Source: BMC Bioinformatics. 2017 Mar 21;18:184. doi: 10.1186/s12859-017-1599-7 (PMC5361736; doi:10.1186/s12859-017-1599-7)
Supplement: Supplementary file 8 — KNN MDR user’s guide. (PDF 116 kb) [file 12859_2017_1599_MOESM8_ESM.pdf]

KNN_MDR: user's guide

Introduction

KNN_MDR is a fortran 90 software implementing the KNN_MDR methodology detailed in (Al Chamlat S. and Farnir F., 2014). The aim of the program is to help obtaining clues about the position of the genes involved jointly in a phenotype. One of the interests of the approach is that it is able to find interating genes even in the absence of marginal effects. This capability, which was already present in other methods, such as MDR (Multi Dimensional Reduction) is made available through KNN_MDR for situations with more markers and more complicated interaction patterns than was feasible computer-wise with "simple" MDR. The current version has been written for binary (0/1) traits and for SNP data, but could easily be extended to other traits/attributes. These options will be included in future versions of the software.

Methodology

This section shortly summarizes how the method works in order to understand the parameters that need to be provided to the software to obtain results. More details can be found in the original publication. Each data point is represented through a phenotype (0/1, where the meaning of these codes is problem-dependent) and a set of N attributes. As mentioned above, in the current version of the program, the attributes correspond to SNP genotypes. It will be assumed that these genotypes are available (through direct genotyping or through an imputation method) for all individuals. The idea behind the MDR methods is to reduce the very large multidimensional space faced in situations involving multiple loci (such as genetic interactions) to one-dimensional space. In basic MDR, a status (0/1) is associated to each multi-locus genotype through a majority vote performed on the individuals presenting this multi-locus genotype in the training set; after that training stage, status (0/1) can be allocated to individuals from the test set on the basis of their multi-locus genotype as well (provided similar genotypes were present in the training set). Accuracy of allocation can then be obtained by computing the false positive (i.e. 1) and false negative (i.e. 0) rates in both training and test sets.
KNN_MDR uses such a strategy. The difference with the basic MDR is that the allocation phase is performed through a K nearest-neighbors approach: a status is allocated on the basis of the most prevalent status within the set of the K nearest neighbors of the tested individual. The neighborood is defined using a distance, which, in the current version, is a simple euclidian distance between

the involved genotypes of both individuals for which a distance is computed. The advantage of such an approach is double: the distance can be easily (i.e. with not much effort computer-wise) computed for any number of markers, and the allocation procedure works even in situation where no other individual in the training set has the same multi-locus genotype. Note that both these points become more relevant as the number of involved markers increases, a practically frequent situation.
An issue exists over the definition of the training and test sets. Again, KNN_MDR mimicks the approach followed in basic MDR using cross-validation: the complete dataset is randomly split into V equally sized subsets, and each subset is sequentially considered as a test set, while the (V-1) other sets are used as training sets. Accuracy is computed for every configuration, and the final model accuracy is computed as the average of the obtained accuracies. In order to balance the true positive and true negative rates in the results, we used "balanced accuracy" as our accuracy measurement, where "balanced accuracy" is defined as the average of true positive and true negative rates.
When looking for sets of genes involved in a phenotype, various attributes sets are usually tested in order to find the one best explaining the data, which is, in our approach, the one with the highest balanced accuracy on the test set. This "best" attribute set will be considered as our "best model".
The last problem is to test the significance of the best model. This is done in our software through a permutation procedure: if a specific attribute set is associated to the phenotype, disrupting the association between phenotypes and genotypes should destroy this association. Consequently, by permuting randomly the phenotypes with respect to the genotypes, we create datasets where no association should exist, which corresponds to the null hypothesis we want to test. Comparing the truly obtained balanced accuracy to the ones obtained on the permuted datasets allows one to obtain an estimation of the p-value associated to our best-model.

Parameters

Several parameters have been definend in the previous and can be transmitted to the program. These parameters are provided through a parameters file, which is invoked while calling the program, as follows:

path/knn_mdr <analysis_name>

In this command, "path" represents the eventual path leading to the executable, and "name" represents the name of the analysis. This name is used to provide the parameters file just discussed (named <analysis_name>.prm) and to name output files (see below). The parameters file is a text file, where each line is used to specify the various options of the program. These options are:

- **ATT_SET_FILE file**: this option is used to specify the file containing the list of attributes sets for which an evaluation is demanded. The best model will be chosen among these attributes sets. Attributes sets are specified on distinct lines of the file by providing a comma separated list

of the positions of the attributes to be considered in the attributes file. When several consecutive attributes have to be used, the notation using the first and the last attribute separated with an hyphen can be used. For example, "1,3,7-10" means "use first, third, seventh, eighth, nineth and tenth attributes" of the attributes file. No default exists for this parameter.

- **ATTRIB_FILE file**: with this option, the file containing all attributes for all individuals in the analysis can be given. Again, "file" is a text file, with one line per individual, and at least as many blank separated columns as the number M of attributes. The attributes file also contains a column with an individual identifier, and may also contain the (0/1) phenotype. Since attributes, in the current version, are SNP genotypes, these genotypes are assumed to be recoded genotypes: for each SNP, one of the allele is arbitrarily considered as the reference allele, and the recoded genotype is simply the number of occurrences of the reference allele in the genotype. Consequently, the allowed attributes values are either 0, 1 or 2. No default exists for this parameter.

- **HELP**: this option is used to obtain an short reminder of the available options.

- **KLOW n**: this option allows to specify the minimum number of neighbors to be used to allocate status to tested individuals. Default is KLOW = 5.

- **KHIGH n**: this option allows to specify the maximum number of neighbors to be used to allocate status to tested individuals. Default is KHIGH = 5.

- **MODEL model**: with this option, a model can be specified. In the current version, the only available model is KNN.... Default is 'KNN'.

- **NB_ATTRIB n**: this options indicates how many attributes should be found in the data file. Default is NB_ATTRIB = 1.

- **NB_CROSS_V n**: this is used to provide the number V of cross-validation subsets. Default is NB_CROSS_V = 10.

- **NB_INDIV n**: this option indicates how many individuals should be found in the data file. Default is NB_INDIV = 1.

- **NB_PERM n**: with this option, the number of permutations can be provided. Default is NB_PERM = 0.

- **PHENO_FILE file**: with this option, the file containing the phenotypes for all individuals in the analysis can be given. As above, "file" is a text file, with one line per individual, one column with the 0/1 phenotype and a column with an individual identifier. this file may be the same as the attributes file. No default exists for this parameter.

- **POS_FIRST_ATTRIB n**: with this option, the position (column number) of the first attribute to be considered can be provided. Default value is POS_FIRST_ATTRIB = 1.

- **POS_LAST_ATTRIB n**: with this option, the position (column number) of the last attribute to be considered can be provided. Default value is POS_LAST_ATTRIB = 1.
- **POS_ID_ATTRIB n**: this option allows to provide the position (column number) of the individual identifier in the attributes file. Default is POS_ID_ATTRIB = 1.
- **POS_ID_PHENO n**: this option allows to provide the position (column number) of the individual identifier in the phenotypes file. Default is POS_ID_PHENO = 1.
- **POS_PHENO n**: this option allows to provide the position (column number) of the phenotype field in the phenotypes file. Default is POS_PHENO = 2.
- **SEED s**: since random choices (cross-validation subsets, permutations) are made, successive invocations of the program will not necesssarily result in identical outputs. Identical (different) runs can be performed by specifying identical (different) seeds through this option.

Example

In this section, we show the use of the program on a simulated example. Twenty attributes are measured for 500 cases and 500 controls. All data are included in one file, named 'sample.dat'. The individual identifier is the first field, followed by the 0/1 phenotype, and then by 20 attributes. An interaction has been introduced artificially between attributes 4 and 12 as follows: all genotypes at locus 4 are generated randomly, irrespectively of the status of the individuals. This should ensure that no marginal effect exists for this locus on the trait. Controls genotypes for locus 12 are also randomly allocated, but cases genotypes for locus 12 are copies of controls ones. This creates an interaction between these two loci.

Attributes and phenotype file

As mentioned, the attributes and the phenotype are included in the same file, named *sample.dat*. The first and last 2 lines are provided below as examples of data lines:

```
1 0 1 1 2 1 2 1 1 1 0 2 2 1 1 1 2 2 1 1 1 1
2 0 1 2 1 1 2 1 0 2 1 1 1 0 1 1 0 0 1 2 1 1
...
999 1 0 0 0 1 0 0 1 2 1 0 0 1 0 0 0 0 0 1 2 2
1000 1 2 1 1 1 1 0 0 0 2 0 1 1 0 1 1 1 1 2 2 1
```

Attributes sets file

The attributes sets file *sample.set* has been created to span the 20 attributes using 5 markers-wide windows. So 4 single windows are first tested (markers 1-5, 6-10, 11-15 and 16-20). After testing single windows, all sets of 2, 3 or 4

windows are considered. This leads to the following file:

```
1-5
6-10
11-15
16-20
1-10
1-5,11-15
1-5,16-20
6-15
6-10,16-20
11-20
1-15
1-10,16-20
1-5,11-20
6-20
1-20
```

Parameters file

The parameters file *sample.prm* is as follows:

```
ATT_SET_FILE sample.set
ATTRIB_FILE sample.dat
KLOW 4
KHIGH 5
MODEL KNN
NB_ATTRIB 20
NB_CROSS_V 10
NB_INDIV 1000
NB_PERM 100
PHENO_FILE sample.dat
POS_FIRST_ATTRIB 3
POS_LAST_ATTRIB 22
POS_ID_ATTRIB 1
POS_ID_PHENO 1
POS_PHENO 2
SEED 123
```

Running the program

To execute a run of the program, simply type:

knn_mdr sample

The program starts running and show intermediate results on the screen. All reported results and more) are also reported to *knn_mdr.log* file for further reference if needed.

Interpreting the output

Three output files are generated: <analysis_name>.log, <analysis_name>.cv and <analysis_name>.perm. Let's first take a look at the 3 first and last lines of *<analysis_name>.cv*. This file shows how the various individuals in the dataset have been allocated to the cross-validation subsets:

```
Indiv    1 -> Subset   1
Indiv    2 -> Subset   2
Indiv    3 -> Subset  10
...
Indiv  998 -> Subset  10
Indiv  999 -> Subset   9
Indiv 1000 -> Subset   5
```

The next file is the *<analysis_name>.perm* file which shows the individual (i.e. for each cross-validation subset) and average (over the cross-validation subsets) balanced accuracies obtained for the real and permuted data. Again we show the few first and last lines of the file:

```
  0          1  0.61317408      0.57258689
  0          2  0.59042907      0.61969697
  0          3  0.57969087      0.56854343
  0          4  0.59012622      0.59803927
  0          5  0.56991446      0.65815413
  0          6  0.59432721      0.53693694
  0          7  0.57333332      0.55000001
  0          8  0.58455396      0.53030300
  0          9  0.58904111      0.65914893
  0         10  0.58518159      0.61642408
  0 AVG  0.58697718      0.59098333
  1          1  0.49010193      0.51542205
  1          2  0.51209480      0.52012885
  1          3  0.51317942      0.50740135
  1          4  0.51018775      0.50146198
  1          5  0.48608297      0.51298702
...        ...         ...             ...
100          5  0.51373267      0.47380954
100          6  0.49153537      0.45833331
100          7  0.50000000      0.47355768
100          8  0.50441492      0.57211542
100          9  0.52405810      0.48684210
100         10  0.50578344      0.46470588
100 AVG  0.49897560      0.50332409
```

The first column represents the permutation number (permutation 0 corresponds to the real not permuted data), the second represents the cross-validation subset (AVG represents the average over all the subsets), the third and the fourth are the training and test balanced accuracies, respectively. Finally, the third file(*<analysis_name>.log*) is the most important one, providing details on the execution of the program along with the main results. The content is given below:

```
Starting the program...
Time is 15:41:05 on January   09,2014
Step 0: name of the analysis: sample
Step 1: obtaining parameters
  KNN_MDR will be launched with following options:
   NB_INDIV                   1000
   NB_ATTRIB                    20
   ATTRIB_FILE         sample.dat
   ATT_SET_FILE        sample.set
   PHENO_FILE          sample.dat
   POS_ID_ATTRIB                 1
   POS_ID_PHENO                  1
   POS_FIRST_ATTRIB              3
   POS_LAST_ATTRIB              22
   POS_PHENO                     2
   NB_CROSS_V                   10
   NB_PERM                     100
   MODEL               KNN
   KLOW                          4
   KHIGH                         5
   SEED                        123
Step 2: reading data
  =>          1000 phenotypic values have been read
  =>          1000 attributes sets have been read
Step 3: defining cross-validation subsets
  => Allocation of CV subsets reported to sample.cv
Step 4: defining attributes sets
  => Number of attributes sets:   15
Step 5: looping through attributes sets
  ==> Attribute set    1:     1,2,3,4,5
  ===> Average balanced accuracy for set            1  =   0.50148523      0.50684011
  ===>  Best balanced accuracy after set            1  =   0.50148523      0.50684011
  ==> Attribute set    2:     6,7,8,9,10
  ===> Average balanced accuracy for set            2  =   0.49123150      0.50559229
  ===>  Best balanced accuracy after set            2  =   0.50148523      0.50684011
  ==> Attribute set    3:    11,12,13,14,15
  ===> Average balanced accuracy for set            3  =   0.47580791      0.49732471
  ===>  Best balanced accuracy after set            3  =   0.50148523      0.50684011
  ==> Attribute set    4:    16,17,18,19,20
  ===> Average balanced accuracy for set            4  =   0.49637920      0.50071424
  ===>  Best balanced accuracy after set            4  =   0.50148523      0.50684011
  ==> Attribute set    5:     1,2,3,4,5,6,7,8,9,10
  ===> Average balanced accuracy for set            5  =   0.50194442      0.52629578
  ===>  Best balanced accuracy after set            5  =   0.50194442      0.52629578
  ==> Attribute set    6:     1,2,3,4,5,11,12,13,14,15
  ===> Average balanced accuracy for set            6  =   0.66471612      0.67562711
  ===>  Best balanced accuracy after set            6  =   0.66471612      0.67562711
  ==> Attribute set    7:     1,2,3,4,5,16,17,18,19,20
  ===> Average balanced accuracy for set            7  =   0.51523346      0.52965009
  ===>  Best balanced accuracy after set            7  =   0.66471612      0.67562711
```

```
  ==> Attribute set    8:     6,7,8,9,10,11,12,13,14,15
  ===> Average balanced accuracy for set           8  =   0.51154667      0.51678431
  ===>  Best balanced accuracy after set           8  =   0.66471612      0.67562711
  ==> Attribute set    9:     6,7,8,9,10,16,17,18,19,20
  ===> Average balanced accuracy for set           9  =   0.49743909      0.51876813
  ===>  Best balanced accuracy after set           9  =   0.66471612      0.67562711
  ==> Attribute set   10:    11,12,13,14,15,16,17,18,19,20
  ===> Average balanced accuracy for set          10  =   0.50408757      0.50820243
  ===>  Best balanced accuracy after set          10  =   0.66471612      0.67562711
  ==> Attribute set   11:     1,2,3,4,5,6,7,8,9,10,11,12,13,14,15
  ===> Average balanced accuracy for set          11  =   0.63244808      0.63826907
  ===>  Best balanced accuracy after set          11  =   0.66471612      0.67562711
  ==> Attribute set   12:     1,2,3,4,5,6,7,8,9,10,16,17,18,19,20
  ===> Average balanced accuracy for set          12  =   0.50658184      0.52353173
  ===>  Best balanced accuracy after set          12  =   0.66471612      0.67562711
  ==> Attribute set   13:     1,2,3,4,5,11,12,13,14,15,16,17,18,19,20
  ===> Average balanced accuracy for set          13  =   0.62913483      0.64112699
  ===>  Best balanced accuracy after set          13  =   0.66471612      0.67562711
  ==> Attribute set   14:     6,7,8,9,10,11,12,13,14,15,16,17,18,19,20
  ===> Average balanced accuracy for set          14  =   0.51725930      0.52325708
  ===>  Best balanced accuracy after set          14  =   0.66471612      0.67562711
  ==> Attribute set   15:     1,2,3,4,5,6,7,8,9,10,11,12,13,14,15,16,17,18,19,20
  ===> Average balanced accuracy for set          15  =   0.58697718      0.59098333
  ===>  Best balanced accuracy after set          15  =   0.66471612      0.67562711
 Step 6: reporting the most significant attributes set
  p-value = 0.0000
  List of attributes: 1,2,3,4,5,11,12,13,14,15
 Now ending the program...
 Time is 15:45:20 on January   09,2014
```

The content of this file is easily understandable. Step 6 reports that set 6, containing markers 1-5 and 11-15, is significantly associated to the phenotype, which is good news given the way the dataset has been generated... Note also that the p_value equal to 0 is obtained through permutations and is only an estimator of the true one. Computing a confidence interval for this p_value would lead to show that p is within $[0; 0.036]$ with a 95% confidence level, so this seems to be a really significant signal!
